# Encoding in a social feedback context enhances and biases behavioral and electrophysiological correlates of long-term recognition memory

**DOI:** 10.1038/s41598-022-07270-9

**Published:** 2022-02-28

**Authors:** Sebastian Schindler, Ria Vormbrock, Johanna Kissler

**Affiliations:** 1 Department of Psychology, Bielefeld University, 33501 Bielefeld, Germany; 2 Institute of Medical Psychology and Systems Neuroscience, University of Münster, Münster, Germany; 3 Otto Creutzfeldt Center for Cognitive and Behavioral Neuroscience, University of Münster, Münster, Germany; 4 Center for Cognitive Interaction Technology (CITEC), Bielefeld University, Bielefeld, Germany

**Keywords:** Long-term memory, Human behaviour, Social behaviour, Social neuroscience

## Abstract

Encoding often occurs in social contexts, yet research has hardly addressed their role in verbal memory. In three experiments, we investigated the behavioral and neural effects of encoding context on memory for positive, negative, and neutral adjectives, contrasting a social-feedback group (*N* = 24) with an explicit verbal-learning (*N* = 24) and a levels-of-processing group (*N* = 24). Participants in the social-feedback group were not aware of a recognition session one week later, but their memory was better than the explicit learning or the levels-of-processing groups'. However, they also exhibited the strongest response bias, particularly for positive words. Brain event-related potentials (ERPs) revealed largest early negativities (EPN) and late positivities (LPP) in the social-feedback group. Only in the subsequent slow-wave did the explicit learning group show higher amplitudes than the other two groups, suggesting reliance on strategic rather than automatic processes. Still, context-driven incidental encoding outweighed explicit instructions, specifying a decisive role of social factors in memory.

## Introduction

Humans are a social species. Therefore, human memory formation, as much other cognitive activity, often occurs in social contexts. Yet, scientific memory research has paid limited attention to the social situatedness of memory encoding, taking memory operations devoid of contextual embedding as its point of departure^1^, thereby delineating many key principles. Meanwhile, contextual binding in space and time has proven crucial for successful episodic memory formation^2^. The social encoding context has, by comparison, received little attention, although recently, social influences on memory systems have attracted scientific interest. These range from investigations into brain mechanisms of social memory conformity^3^ to the finding that the hippocampus, a critical region for spatial navigation and episodic memory formation, also represents a "social space" of relationships^4^. Such findings lend momentum to the hypothesis that the social context in which items are encoded affects their subsequent remembering.

Memory research also initially focused on content-general mechanisms. However, human memory differs depending on the to be encoded material and several content-related factors have been shown to affect episodic memory. For instance, ample evidence indicates that emotionally relevant material is often remembered differently and often better than neutral material^5,6^, which may be due to both higher accuracy and more pronounced response bias for emotional contents^7,8^. Moreover, such emotion effects may be driven by arousal, equally affecting positive and negative contents, or by positive or negative valence^5,9^.

Next to emotional significance, the material's self-reference also plays an important role^10,11^. Whereas emotional significance is typically evaluated via normative ratings, self-reference is typically experimentally induced by a corresponding processing instruction on individually presented words, which is known to enhance recognition or recall. This "self-reference effect" is attributed to elaborative stimulus processing and deep encoding^12^, similar to other levels of processing manipulations, such as concreteness decisions^13^. Setting self-referential processing apart from other encoding manipulations, recent theories propose that the "self" acts as a hub, enabling efficient integration and storage of self-relevant information^11^. Self-referential processing in social contexts is so far hardly understood. However, being confronted with social evaluations from somebody else likely creates a powerful social context, inducing self-referential updating to detect discrepancies with one's self-concept, adapt it, or reject the information [see^14^]. In healthy people, such updating processes are often positively-biased, inducing more elaborative processing of self-serving (rather than merely self-relevant) information^15^. Self-reference and emotion appear to interact and engage at least partly overlapping neural systems^16^.

On the neural level, event-related brain potentials have been used to study mechanisms of stimulus encoding under various processing demands, ranging from free viewing and incidental encoding^17^, over levels of processing instructions^18^, and stimulus appraisals^5^, to self-referential processing^19^ or processing in evaluative social contexts^20^. Early posterior negative (EPN) potentials appearing over the visual cortex around 200 ms post-stimulus generally index feature-based attention and conceptual stimulus encoding, which can gate episodic memory encoding^21^. EPN potentials have been found to co-vary with task-driven attention deployment^22^, emotionally motivated attention^23^, self-reference^19^, but also contextually induced social relevance^20^. A series of later surface-positive potentials occurring over frontal and parietal brain areas index episodic memory encoding proper [e.g., see^24^]. The parietal part of these effects, typically known as late positive potential (LPP) and occurring from about 400 ms post stimulus-onset, is also sensitive to explicit emotional appraisal^5^ and self-reference^19^. Moreover, higher late positive potentials during emotional appraisal predict better subsequent memory for the respective material^5^. Whereas P3b-like late parietal positivities can vary in topography and functional significance, they often have overlapping generators, at least some of which are likely to reflect memory formation^25,26^. Fronto-parietal slow waves after about 800 ms are generally assumed to reflect strategic verbal memory encoding^27^.

Our own previous work showed that social-evaluative context considerably impacts amplitudes of both early negative and late positive brain potentials. When identical trait adjectives were presented as personality feedback in virtual interaction setups, EPN and LPP brain potentials were considerably larger when feedback seemed to come from more relevant interaction partners^20,28^. However, no previous studies investigated whether the putative social-evaluative context in which material was presented had any long-term memory consequences.

The present research aims to fill this gap, investigating the influence of perceived social context on memory formation. We study the effects of a social-evaluative encoding context, which previously yielded large electrophysiology effects, on long-term recognition memory of positive, negative, and neutral trait adjectives. We present the exact same word lists in three different encoding contexts, measuring event-related brain potentials at encoding and recognition memory one week later. In detail, we compare a group that incidentally encoded words in a social-evaluative feedback context with two other encoding groups. As in previous work^20,28–30^ the social-evaluative feedback context was established by asking participants to introduce themselves briefly in front of a camera and telling them that they would be later evaluated on the basis of their self-introduction, either by another person next door or by a computer system. To disclose the evaluations, the participants would later see trait adjectives on a computer screen and given the opportunity to agree or disagree with the evaluations. In a second group, participants were instructed to learn the same trait adjectives intentionally, the material being divided into two blocks, and in a third group, participants incidentally encoded the words via a levels-of processing manipulation. The levels-of-processing manipulation also consisted of two blocks, comprising self-referential processing, where participants could decide whether they found an adjective self-descriptive and a concreteness/abstractness decision without self-reference. Behaviorally, we expected self-reference judgments to be positively biased, with participants accepting positive adjectives more often as feedback (Experiment 1) and judging them more often as self-descriptive (Experiment 3) than negative or neutral adjectives. We also expected recognition memory to be better for emotional (in the social context and the self-reference condition particularly positive) than for neutral adjectives. On the electrophysiology level, we expected EPN and LPP amplitudes to be enhanced by emotional content as well as social context. Specifically, we investigated how the different encoding contexts, namely the social feedback manipulation, the learning instruction, and the levels-of-processing tasks, compared regarding recognition memory and EPN, LPP, and positive slow wave amplitudes.

## Results

### Behavior during the encoding task

#### Social-feedback group (feedback acceptance)

Positive feedback was more often accepted than negative and neutral feedback (*ps* < 0.001), while neutral feedback was also more often accepted by participants than negative feedback (*p* = 0.001) as reflected by a main effect of emotional content (*F*_(2,46)_ = 62.99, *p* < 0.001, η_p_^2^ = 0.733) and subsequent follow-up tests. A smaller main effect of feedback sender (*F*_(1,23)_ = 5.12, *p* = 0.033, η_p_^2^ = 0.182) revealed that participants were more likely to accept feedback from the more relevant (“human”) sender. No interaction was found (*F*_(2,46)_ = 0.48, *p* = 0.622, η_p_^2^ = 0.020). Reaction times were 913 ms on average. While they did not differ significantly between sender conditions (*F*_(1,19)_ = 4.09, *p* = 0.057, η_p_^2^ = 0.177), they were descriptively longer for decisions on human feedback (999 ms) compared to computer feedback (827 ms). There were no significant differences between emotion conditions and no interaction (*Fs* < 0.81, *ps* > 0.452).

#### Levels-of-processing group (self-descriptiveness and concreteness)

Here, effects of task (*F*_(1,23)_ = 21.22, *p* < 0.001, η_p_^2^ = 0.480), emotion (*F*_(2,46)_ = 16.25, *p* < 0.001, η_p_^2^ = 0.414), and their interaction were observed (*F*_(2,46)_ = 23.31, *p* < 0.001, η_p_^2^ = 0.503). Within the self-reference task participants selected significantly more positive than both neutral (*p* < 0.001) and negative (*p* < 0.001) words as self-descriptive, neutral and negative words not differing (*p* = 1.0), resulting in a large emotion effect (*F*_(2,46)_ = 30.61, *p* < 0.001, η_p_^2^ = 0.571). In the concreteness task, there was no effect of emotional content on concrete/abstract decisions (*F*_(2,46)_ = 1.05, *p* = 0.359, η_p_^2^ = 0.044). Reaction times were 1110 ms on average and differed between conditions (*F*_(1,23)_ = 12.76, *p* = 0.002, η_p_^2^ = 0.357), with longer reaction times during concreteness decisions (1258 ms) than during self-reference decisions (963 ms). There were no differences between emotion conditions and no interaction (*Fs* < 0.23, *ps* > 0.797).

### Recognition memory (one week later)

**Figure 1:**
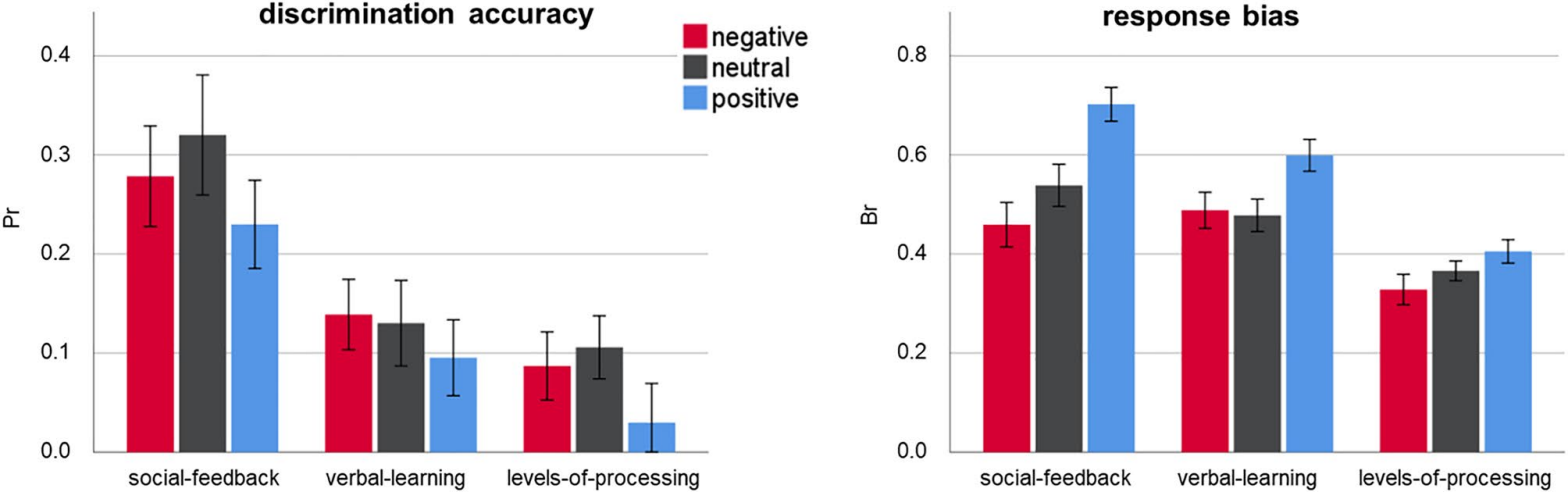
Behavioral data for the social-feedback, verbal-learning, and levels-of-processing group. Discrimination accuracy (P_r_ = Hits – False alarms) and response bias (B_r_ = false alarms/(1-P_r_)) are shown per emotional category and group. Error bars depict ± 1 Standard Error of the Mean.

Recognition memory data are shown in Fig. 1 and further detailed in Table 1 and Supplement A.

**Table 1:** Memory data for the three groups.

Social-feedback group (*N* = 24)									
	Condition *F*_(1,23)_	Emotion *F*_(2,46)_	Interaction *F*_(2,46)_	Human feedback			Computer feedback		
				Negative	Neutral	Positive	Negative	Neutral	Positive
P_r_	1.12	3.53*	2.17	.32 (0.25)	.35 (0.30)	.24 (0.21)	.24 (0.29)	.29 (0.35)	.22 (0.27)
B_r_	3.00	47.70***	4.58*	.50 (0.25)	.57 (0.23)	.71 (0.17)	.44 (0.22)	.48 (0.25)	.72 (0.19)

Verbal-learning group (*N* = 21)									
	Condition *F*_(1,20)_	Emotion *F*_(2,40)_	Interaction *F*_(2,40)_	Block A			Block B		
				Negative	Neutral	Positive	Negative	Neutral	Positive
P_r_	0.07	0.83	0.57	.13 (0.15)	.14 (0.20)	.09 (0.19)	.14 (0.20)	.13 (0.22)	.10 (0.18)
B_r_	0.47	23.48***	0.09	.48 (0.17)	.48 (0.15)	.60 (0.15)	.50 (0.17)	.49 (0.17)	.61 (0.16)

Levels-of-processing group (*N* = 23)									
	Condition *F*_(1,22)_	Emotion *F*_(2,44)_	Interaction *F*_(2,44)_	Self-reference task			Semantic task		
				Negative	Neutral	Positive	Negative	Neutral	Positive
P_r_	0.04	3.53*	30.06***	.13 (0.19)	.00 (0.22)	.11 (0.20)	.05 (0.21)	.21 (0.18)	−.05 (0.25)
B_r_	1.39	8.75**	6.80**	.36 (0.18)	.33 (0.09)	.45 (0.16)	.31 (0.14)	.40 (0.20)	.38 (0.11)

Significant values are in bold.

* = *p* < 0.05, ** = *p* < 0.01, *** = *p* < 0.001. Standard deviations appear in parentheses. P_r_ = discrimination accuracy, B_r_ = recognition bias.

### Discrimination accuracy

As shown in Fig. 1, left panel, experimental groups differed in discrimination accuracy (*F*_(2,65)_ = 7.57, *p* = 0.001, η_p_^2^ = 0.189). The social-feedback group was significantly more accurate than both the verbal-learning (*p* = 0.007) and the levels-of-processing group (*p* < 0.001). The latter two groups did not differ (*p* = 0.403). Group and emotion did not interact (*F*_(4,130)_ = 0.45, *p* = 0.776, η_p_^2^ = 0.014), but a three-way interaction between group, encoding condition, and emotion (*F*_(4,130)_ = 20.53, *p* < 0.001, η_p_^2^ = 0.387) indicated that depending on the experiment, emotion effects differed between conditions (see Table 1). Within the social feedback group, discrimination accuracy did not differ between encoding during human compared to computer feedback for negative (*p* = 0.120), neutral (*p* = 0.330), or positive words (*p* = 0.774). Likewise, for the verbal-leraning group, no encoding differences between block A and block B were found for all three emotion conditions (*ps* > 0.441). Within the levels-of-processing group, encoding in the self-reference task compared to the concreteness task led to higher accuracy for positive words (*p* = 0.003) but lower accuracy for neutral words (*p* = 0.001). For negative words, accuracy did not differ between the self-referential and concreteness tasks (*p* = 0.113).

### Response bias

Response bias differed between the groups (*F*_(2,65)_ = 10.31, *p* < 0.001, η_p_^2^ = 0.241; see Fig. 1, right panel). Both the social-feedback and the verbal-learning group responded more liberally than the levels-of-processing group (*p* < 0.001 and *p* = 0.002, respectively). Group interacted with emotion (*F*_(4,130)_ = 9.09, *p* < 0.001, η_p_^2^ = 0.218; see Table 1), suggesting specific bias differences between the groups. We compared the bias per emotion category, showing group differences for positive words (*F*_(2,65)_ = 25.367, *p* < 0.001, η_p_^2^ = 0.438), with a larger positive-bias in the social feedback group compared to the verbal-learning (*p* = 0.020) and levels-of-processing group (*p* < 0.001; see Fig. 1, right panel). There was also a more liberal responding to positive words in the verbal-learning than in the levels-of-processing group (*p* < 0.001). Response bias differed also for neutral words (*F*_(2,65)_ = 7.03, *p* = 0.002, η_p_^2^ = 0.178), showing no difference between the social feedback and verbal-learning group (*p* = 0.208), but more conservative responding in the levels-of-processing group compared to the other two groups (*ps* < 0.05). Finally, response bias group differences were also found for negative words (*F*_(2,65)_ = 4.94, *p* = 0.010, η_p_^2^ = 0.132), again showing no difference between the social feedback and verbal-learning group (*p* = 0.595), but more conservative responding in the levels-of-processing group compared to the other two groups (*ps* < 0.05). Finally, there was a three-way interaction between group, condition, and emotion concerning response bias (*F*_(4,130)_ = 6.42, *p* = 0.001, η_p_^2^ = 0.165). Follow-up investigations within groups revealed for the social-feedback no significant bias differences between encoding words in the human or computer block for positive (*p* = 0.882) and negative words (*p* = 0.053), but more liberal responding to neutral words encoded as human feedback (*p* = 0.032). For the verbal-learning group, bias did not differ between the three emotion conditions (*ps* > 0.288). For the levels-of-processing group, encoding words in the self-relevance compared to the concreteness task led to more liberal responding to positive (*p* = 0.004) and negative words (*p* = 0.019), but not for neutral words (*p* = 0.126).

## Encoding ERPs

### EPN (200–400)

**Figure 2:**
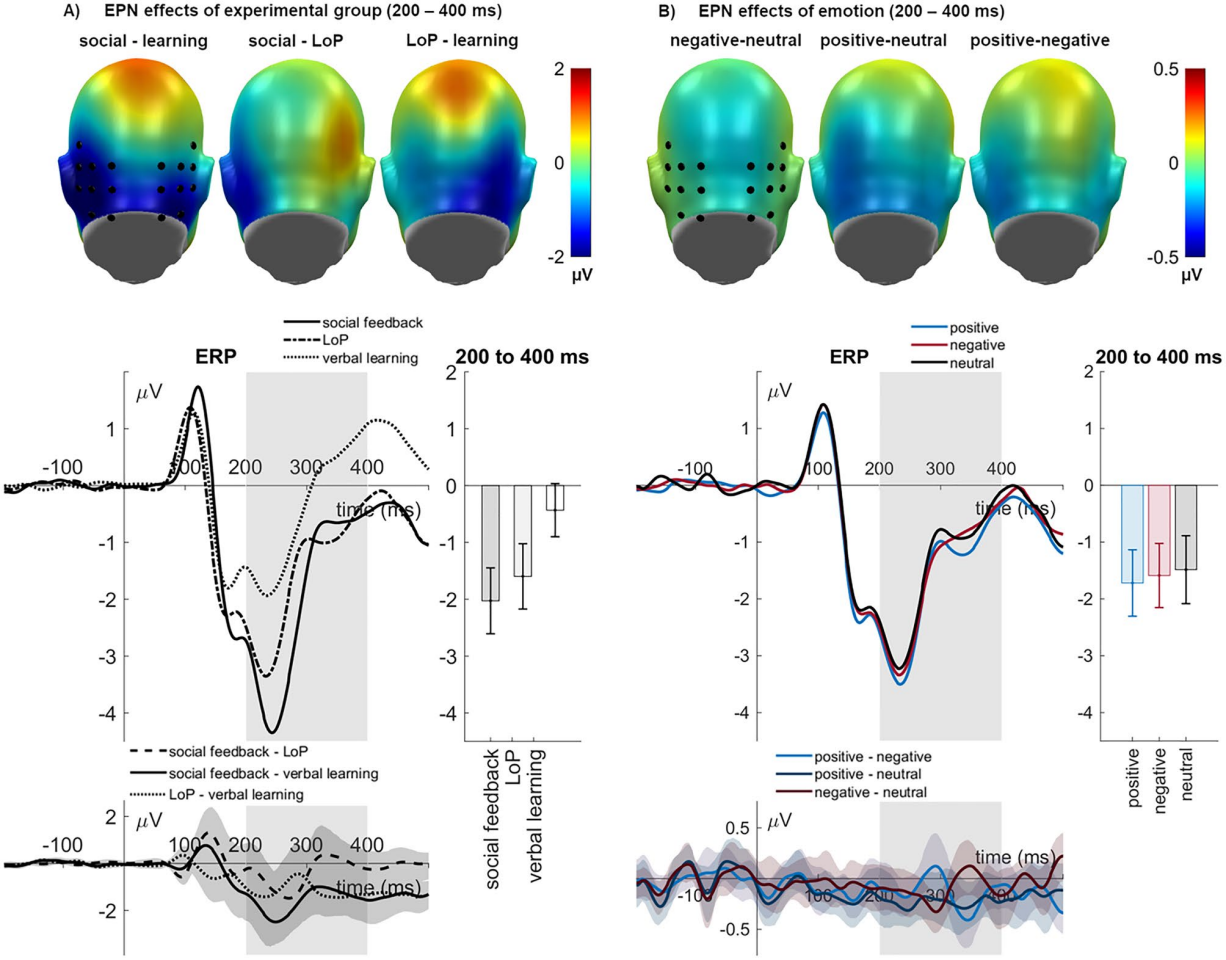
EPN effects of (**A**) experimental group and (**B**) emotion. Scalp topographies depict the mean amplitude differences for the respective interval. ERPs show the time course averaged from highlighted sensors. Between-group difference potentials contain 95% bootstrap confidence intervals. Between-emotion difference-potentials contain 95% bootstrap confidence intervals of intra-individual differences. For bar charts, error bars show 95% confidence intervals.

For the EPN, an effect of experimental group was found (*F*_(2,69)_ = 8.50, *p* < 0.001, η_p_^2^ = 0.198; see Fig. 2a). The social-feedback group had a larger EPN than the verbal learning (*p* < 0.001), but not than the levels-of-processing group (*p* = 0.286). The latter showed also a larger EPN than the verbal-learning group (*p* = 0.005). Further, there was a main effect of emotion (*F*_(2,138)_ = 6.65, *p* = 0.002, η_p_^2^ = 0.088; see Fig. 2b) that was due to larger EPN for positive (*p* < 0.001) and negative (*p* = 0.020) words compared to neutral ones. Positive and negative words did not differ (*p* = 0.272). No interactions emerged (*Fs* < 0.95*, ps* > 0.438)*.*

### LPP (400–800 ms)

**Figure 3:**
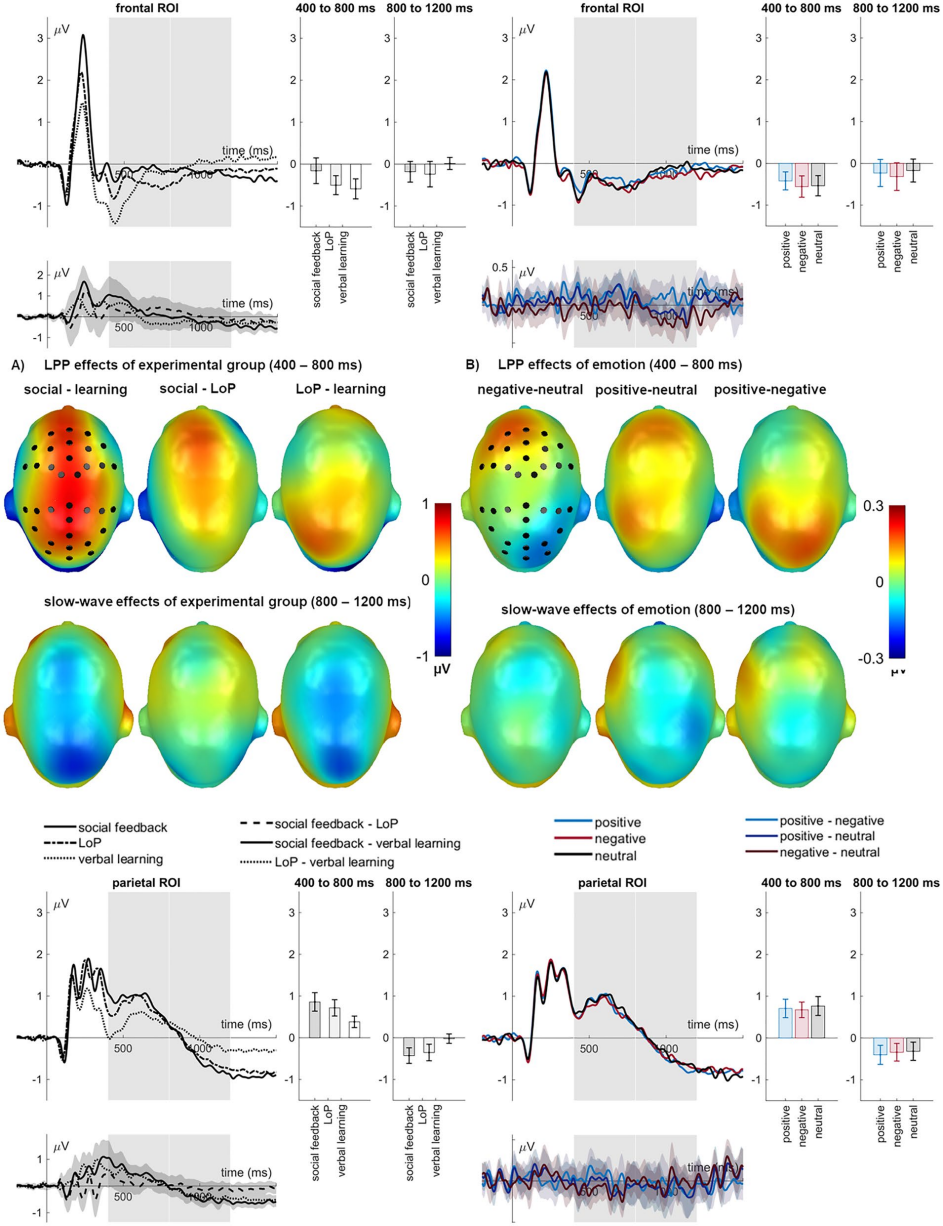
LPP and slow-wave effects of (**A**) experimental group and (**B**) emotion. Scalp topographies depict the mean amplitude differences for the respective interval. ERPs show the time course from averaged highlighted sensors (upper panel frontal ROI, lower panel parietal ROI). Respective difference potentials contain 95% bootstrap confidence intervals of the group differences. Emotion difference waves contain 95% bootstrap confidence intervals of intra-individual differences. For bar charts, error bars show 95% confidence intervals.

Experimental groups differed also for the LPP (*F*_(2,69)_ = 5.65, *p* = 0.005, η_p_^2^ = 0.141; see Fig. 3a). The social-feedback group had much larger LPP amplitudes than the verbal-learning (*p* = 0.001), and somewhat larger ones than the levels-of-processing group (*p* = 0.070), the latter groups did not differ (*p* = 0.134). A main effect of emotion (*F*_(2,138)_ = 3.71, *p* = 0.027, η_p_^2^ = 0.051; see Fig. 3b) showed a larger LPP for positive compared to neutral (*p* = 0.031) and compared to negative words (*p* = 0.019), the latter two not differing (*p* = 0.908). An interaction of emotion and sensor ROI (region of interest) (*F*_(4,138)_ = 4.50, *p* = 0.002, η_p_^2^ = 0.115) revealed that the emotion effect was more pronounced over the parietal ROI (*F*_(2,138)_ = 3.88, *p* = 0.023, η_p_^2^ = 0.103) than over the frontal ROI (*F*_(2,138)_ = 2.53, *p* = 0.083, η_p_^2^ = 0.035). Similar to the global emotion effect, for the parietal cluster a larger LPP for positive than negative words (*p* = 0.007) was found. Neither positive (*p* = 0.180) nor negative words (*p* = 0.156) differed from neutral ones. A further four-way interaction occurred (*F*_(4,138)_ = 3.48, *p* = 0.010, η_p_^2^ = 0.092) which is resolved and illustrated in the Supplement—Section B. Sensor ROIs are further described in the methods section below.

### Slow-wave (800–1200 ms)

Groups also differed in their slow-wave amplitudes (*F*_(2,69)_ = 3.57, *p* = 0.033, η_p_^2^ = 0.094; see Fig. 3a). Here, the verbal-learning group had a larger positive-going slow-wave than the social-feedback (*p* = 0.021) and the levels-of-processing groups (*p* = 0.026), who did not differ (*p* = 0.933). No other main effects (*F*_(2,138)_ = 1.08, *p* = 0.342, η_p_^2^ = 0.015) or significant interactions emerged (*Fs* < 2.10*, ps* > 0.084)*.*

### Run 2/passive viewing re-presentation of word stimuli

After debriefing, a passive viewing run was designed to test for the immediate effects of the preceding experimental setup. There were no effects of the experimental group, emotion, or any interactions between these factors for the EPN, LPP, and the slow waves (*Fs* < 1.40*, ps* > 0.236)*.*

### Correlations between encoding ERPs and memory performance

Correlations showed the strongest relationships between ERPs and memory indices for LPP amplitudes (see Table 2). For the social-feedback group, parietal LPP amplitudes were related negatively to the false alarm rate. Bayes factor analysis revealed moderate evidence of a relationship for positive words. Although the levels-of-processing group showed descriptively a similar relationship pattern over parietal sensors, Bayes Factors did not support such a relationship. For the verbal-learning group, the strongest relationships of LPP amplitudes were found over the frontal cluster. Here, strong evidence was found for a relationship between ERP amplitudes during encoding and hit rate during recognition for neutral words. A similar pattern was found concerning negative and positive words, but Bayes Factors showed only anecdotal evidence of a relationship.

**Table 2:** Relationship of ERPs during encoding and memory performance.

	Frontal LPP (400 – 800)			Parietal LPP (400 – 800)		
	Negative	Neutral	Positive	Negative	Neutral	Positive
Social-feedback group (*N* = 24)						
Hits Pearson *r*	−.156	−.304	−.241	.039	−.113	−.210
*p*-Value^a^	.468	.149	.256	.856	.599	.324
BF_10_	0.325	0.679	0.465	0.257	0.289	0.401
FA Pearson *r*	.028	−.056	.101	−.194	−.317	**−.495**
*p*-Value^a^	.896	.794	.637	.363	.132	**.014**
BF_10_	0.255	0.261	0.381	0.374	0.740	4.403*
Verbal learning group (*N* = 21)						
Hits Pearson *r*	.402	**.703**	.415	−.332	−.077	−.146
*p*-Value^a^	.071	** < .001**	.061	.142	.740	.528
BF_10_	1.251	98.105**	1.399	0.745	0.285	0.326
FA Pearson *r*	.286	.343	.427	.032	−.055	−.046
*p*-Value^a^	.209	.128	.053	.890	.812	.845
BF_10_	0.567	0.801	1.558	0.273	0.278	0.275
Levels-of-processing group (*N* = 23)						
Hits Pearson *r*	−.009	−.101	.267	−.006	.419	.356
*p*-value^a^	.969	.648	.217	.979	.033	.095
BF_10_	0.259	0.285	0.530	0.259	1.678	0.962
FA Pearson *r*	−.093	−.100	.283	−.301	−.420	.139
*p*-value^a^	.671	.651	.191	.162	.046	.0528
BF_10_	0.281	0.285	0.579	0.650	1.685	0.312

	Frontal slow-wave (800–1200)			Parietal slow-wave (800–1200)		
	Negative	Neutral	Positive	Negative	Neutral	Positive
Social-feedback group (*N* = 24)						
Hits pearson *r*	.181	.072	.119	.266	.033	.120
*p*-value^a^	.398	.737	.578	.209	.878	.578
BF_10_	0.355	0.267	0.293	0.533	0.256	0.293
FA pearson *r*	−.125	−.329	−.207	.203	−.186	−.226
*p*-value^a^	.561	.117	.333	.341	.385	.289
BF_10_	0.297	0.809	0.395	0.389	0.362	.431
Verbal learning group (*N* = 21)						
Hits pearson *r*	.024	−.221	.149	−.016	−.125	−.060
*p*-value^a^	.917	.336	.519	.944	.590	.796
BF_10_	0.272	0.417	0.329	0.271	0.310	0.279
FA pearson *r*	.011	−.019	.002	−.037	.262	.162
*p*-value^a^	.962	.934	.992	.873	.251	.482
BF_10_	0.271	0.271	0.270	0.274	0.501	0.341
Levels-of-processing group (*N* = 23)						
Hits pearson *r*	.135	−.124	.032	.104	.123	.059
*p*-value^a^	.539	.572	.886	.636	.575	.790
BF_10_	0.309	0.301	0.261	0.287	0.300	0.267
FA pearson *r*	.010	.111	.108	−.272	−.201	−.024
*p*-value^a^	.964	.613	.625	.209	.359	.913
BF_10_	0.259	0.292	0.289	0.545	0.385	0.260

Significant values are in bold.

LPP and slow wave amplitudes for the respective emotional category were correlated with hits during recognition for this emotional category. Hits and False alarms (FA) are examined. ^a^Bonferroni-corrected significance threshold is *p* < .00417; * = BF_10_ > 3; ** = BF_10_ > 10. (where BF_01_ = 1/ BF_10_).

## Discussion

Humans typically acquire information in social contexts. Therefore, we investigated its role for long-term recognition memory and encoding ERPs for positive, negative, and neutral words. We compared a group for whom a social context was induced via a self-introduction followed by evaluative feedback ("social-feedback" group) with a verbal-learning and a standard levels-of-processing group. Although the social-feedback group was not aware of the recognition session one week later, this group showed superior recognition accuracy, but also an increased response bias, particularly for positive feedback adjectives. Encoding ERPs revealed the highest EPN and LPP amplitudes in the social-feedback group. Only in the slow-wave window were ERPs most positive-going in the verbal-learning group. ERPs from the levels-of-processing group fell in between the other two. Together, these findings reveal a distinct effect of evaluative social context, enhancing long-term recognition memory and encoding ERPs beyond both explicit learning and standard levels of processing manipulations, while also inducing specific biases.

The highest memory accuracy in the social feedback group indicates that the social context facilitated memory encoding, likely via elaborative stimulus processing which is known to enhance memory [e.g., see^11,12,31^]. Since explicit learning tasks are typically easier and result in better memory than incidental ones^32,33^, these findings highlight the power of social context to increase stimulus encoding.

Groups also differed in their response bias, with less conservative responses in the social-feedback and the verbal learning groups. Moreover, the social-feedback group was specifically biased towards classifying positive words as "old". Healthy participants are known to have optimistic feedback expectations^34^, leading to distorted memory when information is incongruent with their positive self-view^35^. In fact, better than expected evaluations induce positively biased self-updating^36^ as well as a specific feedback-related positivity^37^. Self-serving tendencies also occurred in feedback acceptance (social-feedback group) and self-descriptiveness decisions (levels-of-processing group), the highest acceptance rates for positive adjectives corresponding with positive self-views of healthy participants [e.g., see^34^].

Concerning ERPs, words in the social feedback context elicited the largest EPN and LPP amplitudes, followed by levels-of-processing and, finally, instructed learning. Per se, these ERP modulations are broadly in line with findings of increased EPN and LPP amplitudes for various manipulations of self-reference and social feedback^19,38,39^. Given the EPN's role in early attentional selection^22^, results show a rapid prioritizing role of social-evaluative context beyond mere task-relevance. The highest LPP in the social feedback group is also broadly in line with the view that P3b-like components, such as the LPP, index successful memory encoding^25,26^. Dolcos and Cabeza^5^ found that positive and negative items that elicited larger LPP than neutral ones during emotional appraisal were subsequently better remembered. Such items also elicited a larger fronto-central subsequent memory positivity than neutral ones. Whereas we have no subsequent memory ERP data for the present set of experiments, the much larger encoding positivities we found in the social context condition in parallel with the considerably higher recognition rates are certainly consistent with the view that late positive potentials reflect, at least in part, episodic memory encoding, although the subsequent memory effect provides a purer measure for this. Exploratory correlations also support a role for the LPP in memory formation (see Table 2). Topographic differences may indicate that the verbal learning group relied more on frontally mediated strategic mechanisms, particularly to support encoding of neutral items. However, given the modest sample sizes of each group, these relationships should be interpreted cautiously.

Only in the slow-wave window did the verbal-learning group exhibit more positive-going ERPs than the other two groups. Given the role of positive-going slow waves in strategic verbal memory encoding^27^, this finding might explain why the verbal-learning group had similar recognition accuracy (and even more hits) than the levels-of-processing group, despite having smaller amplitudes on the earlier ERPs. Therefore, encoding in the social feedback and levels-of-processing groups seems to have relied more on automatic mechanisms, whereas the verbal-learning group recruited more strategic processes.

As expected, significant emotion effects on brain potentials were also observed, although they were smaller than the context effects. Emotion sensitivity of EPN and LPP components aligns with much previous research [e.g., see^17,40–42^]. Largest amplitudes for positive words have also been reported before [see^43^]. Self-referential processing might have contributed to a more substantial increase for positive content, whereas in other situations, a bias towards negative material might occur [e.g., see^40,41^].

Overall, we show that social context during encoding enhances long-term recognition memory beyond explicit learning or typical deeper incidental encoding tasks (self-descriptiveness or concreteness decisions). Notably, although both the social feedback and self-descriptiveness task probably recruited self-referential processing, effects in the feedback condition, where any self-reference was socially contextualized, by far exceeded those of task-driven self-referential processing alone. This finding may give rise to questions regarding the role of the initial structured interview and self-introduction, which were designed to make the entire social context more salient and credible. However, this manipulation also bears some similarities with common stress induction methods. We observed no specific increases in state anxiety ratings compared to the other groups. Trait anxiety ratings did not differ either. Still, the present implementation might be regarded as a mild form of stress induction and may have induced arousal or led to secretion of stress hormones which we did not measure. The existing literature on memory effects of pre-encoding stress suggests that the present effects are unlikely solely due to pre-encoding stress. Previously reported effects of pre-encoding stress 30 min before encoding (which would be quite similar to the present timing) on recognition memory the next day reveal modest^44^, ambiguous^45,46^, and often also impairing effects^47^ which contrasts with the quite large effects that we found after a weeklong retention interval. Nevertheless, any endocrine effects of the present context induction should be addressed in future studies. Although the present retention interval of a week leaves ample time for consolidation or spontaneous rehearsal, which we cannot directly assess, the immediate repetition run revealed no significant effects, suggesting that effects were either due to initial encoding, where corresponding ERP modulations occurred, or arose considerably later.

The fact that responses in the social feedback and the levels-of-processing groups were self-paced may give rise to concerns regarding processing times. However, processing times were quite similar in these two experiments, and if anything, they were longer in the levels-of-processing group, yet memory performance was much higher in the social feedback group. The levels-of-processing group returned for testing on average half a day after the social feedback group, which might be another source of concern. Yet, there was no difference in testing lag between the verbal learning group and the social feedback group. The verbal learning group was the only group aware of the test, and they could use the interstimulus interval for rehearsal while the other two groups were performing the decision task. Still, the social feedback group clearly outperformed the other two groups regarding recognition memory.

Our focus was on the between-groups analysis, which de-emphasizes potentially interesting effects in individual conditions. Still, in Experiment 1, some "sender" differences occurred at encoding (see Supplement B), replicating previous research [e.g., see^28^]. However, these had little effect on long-term recognition, suggesting that participants integrated both blocks from experiment 1 into one episode whose items were considerably more memorable than those from the other two experiments. Interactions in the levels-of-processing group indicate that encoding task modulates emotion effects in long-term recognition memory, only self-descriptiveness and not concreteness decisions resulting in an emotion effect on recognition accuracy, in line with the suggestion of interacting memory effects of self and emotion^16^.

In sum, although we used identical stimuli and presentation parameters across three experiments, the psychological encoding contexts elicited pronounced between-group differences in recognition memory and ERPs: The social-feedback context enhanced recognition memory beyond both explicit verbal-learning and self-descriptiveness/concreteness judgments. It also induced the largest EPN and LPP amplitudes, followed by levels-of-processing and verbal learning. The most positive-going slow wave amplitudes in the verbal learning group suggest that this group might have relied particularly on strategic memory processes. Our findings specify some important social factors in human long-term memory. They resonate with the fact that humans typically acquire information in social contexts [e.g., see^48^], social evaluation being a particularly salient factor in human life [e.g., see^49^]. Finally, these results have implications for memory formation in educational settings or memory assessment in legal contexts, where the extent to which individuals were exposed to an evaluative social context will affect their memory for elements of the initial episode.

## Methods

### Participants

Three groups of twenty-four participants each were recruited at Bielefeld University. Participants provided written informed consent and received 10 Euros per hour for participation. The Ethics Committee at Bielefeld University approved the study and the study was performed in accordance with the regulations of the Declaration of Helsinki. All subjects were right-handed, had normal or corrected-to-normal vision, and were free from a self-reported neurologic or psychiatric disorder. They were tested twice with a lag of about one week (T1–T2 difference *M* = 7.13 days, *Min* = 6 days, *Max* = 9 days, see Table 3). Experimental groups did not differ concerning BDI scores (*F*_(2,68)_ = 0.03, *p* = 0.973, η_p_^2^ = 0.001), or STAI state (*F*_(2,68)_ = 1.34, *p* = 0.269, η_p_^2^ = 0.038) and trait anxiety scores (*F*_(2,64)_ = 0.72, *p* = 0.492, η_p_^2^ = 0.022). There was a significant effect of the T1-T2 difference (*F*_(2,64)_ = 7.30, *p* = 0.001, η_p_^2^ = 0.186), showing a significant longer latency of about half a day for the levels-of-processing group compared to both the social-feedback group (*p* = 0.004) and verbal-learning group (*p* < 0.001). The social-feedback and verbal-learning groups did not differ (*p* = 0.464). Four participants in total did not attend the second session. One of these was in the levels-of-processing group and three in the verbal-learning group.

**Table 3:** Demographic information for the participants in each experiment.

Variable	Social-feedback group (*N* = 24)	Verbal-learning group (*N* = 24)	Levels-of-processing group (*N* = 24)
Gender female/male	14/10	19/5	18/6
Age	24.13 (2.54)	25.48^a^ (4.71)	24.36^b^ (3.08)
BDI Score*	4.87 (5.31)	4.61^a^ (3.93)	4.91 (5.27)
STAI state**	33.92 (6.97)	35.48^a^ (4.21)	32.54 (6.82)
STAI trait**	36.79 (8.81)	38.57^c^ (6.73)	35.68^b^ (8.10)
T1–T2 difference	7.13 (0.61)	7.00^b^ (0.31)	7.61^a^ (0.66)

(a) information is missing from one (b) missing from two (c) missing from three participants. *BDI: Beck's Depression Inventory [50]. **STAI: State-Trait Anxiety Inventory [51].

### Stimuli

The stimulus set had been rated by 22 other students on nine-point Likert-type scales in terms of valence and arousal using the Self-Assessment Manikin^52^ and a similarly constructed concreteness scale. The selected 270 adjectives (90 negative, 90 neutral, 90 positive) were assigned to three separate lists. Neutral adjectives were allowed to deviate from emotional adjectives on both arousal and concreteness since truly neutral trait adjectives are rare in an interpersonal evaluative context. The three lists did not differ in relevant emotional and lexical properties, and the list-condition assignment was counterbalanced (see Table 4).

**Table 4:** Comparisons of negative, neutral, and positive adjectives for three separate lists by One-Way-ANOVAs.

Variable	Negative adjectives each list (*N* = 30)	Neutral adjectives each list (*N* = 30)	Positive adjectives each list (*N* = 30)
**Valence: Main effect emotion *F***_**(2,270)**_ = 1182.37***, Interaction Emotion*List *F*_(4,270)_ = 1.24			
List 1	2.83^a^ (0.64)	4.58^b^ (0.64)	7.44^c^ (0.72)
List 2	2.78^a^ (0.57)	4.84^b^ (0.50)	7.32^c^ (0.70)
List 3	2.70^a^ (0.56)	4.84^b^ (0.55)	7.27^c^ (0.77)
**Arousal: Main effect emotion *F***_**(2,270)**_ = 121.80***, Interaction Emotion*List *F*_(4,270)_ = 0.25			
List 1	4.95^a^ (0.89)	3.42^b^ (0.98)	4.79^a^ (0.75)
List 2	4.96^a^ (0.71)	3.20^b^ (0.68)	4.71^a^ (0.73)
List 3	4.98^a^ (0.85)	3.20^b^ (0.78)	4.69^a^ (0.63)
**Concreteness: Main effect emotion *F***_**(2,270)**_ = 73.15***, Interaction Emotion*List *F*_(4,270)_ = 0.19			
List 1	3.61^a^ (0.93)	5.32^b^ (1.67)	3.15^a^ (1.22)
List 2	3.62^a^ (0.96)	5.49^b^ (1.36)	3.38^a^ (1.20)
List 3	3.40^a^ (0.91)	5.03^b^ (1.42)	3.17^a^ (1.13)
Word length: Main effect of emotion *F*_(2,270)_ = 0.29, Interaction Emotion*List *F*_(4,270)_ = 0.07			
List 1	8.93^a^ (2.12)	8.97^a^ (1.92)	9.20^a^ (2.48)
List 2	8.93^a^ (2.52)	9.13^a^ (2.57)	9.03^a^ (3.17)
List 3	8.80^a^ (2.63)	9.03^a^ (2.54)	9.30^a^ (2.65)
Frequency (per million): Main effect of emotion *F*_(2,270)_ = 0.11, Interaction Emotion*List *F*_(4,270)_ = 0.01			
List 1	375.03^a^ (482.35)	393.37^a^ (517.83)	344.80^a^ (485.97)
List 2	396.33^a^ (589.39)	397.80^a^ (526.32)	367.07^a^ (490.14)
List 3	359.97^a^ (580.87)	383.83^a^ (535.67)	356.80^a^ (466.75)

*** = *p* ≤ 0.001. Cells provide the means for the different word attributes with standard deviations in parentheses; means for the same List not sharing subscripts differ at *p* ≤ 0.05 based on LSD test post-hoc comparisons.

### Procedure

**Figure 4:**
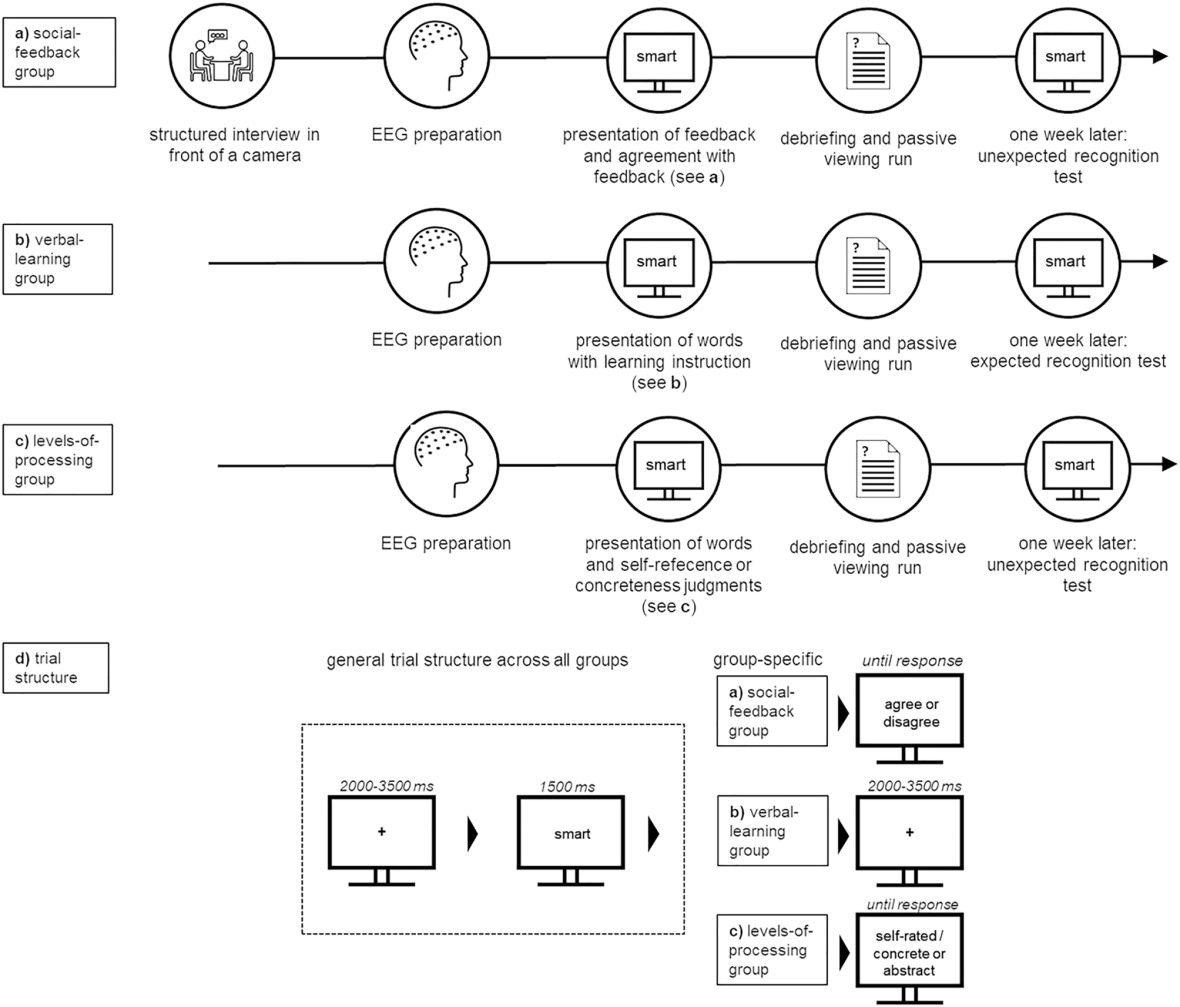
Example experimental and main experiment trial structure for the (**a**) social-feedback group, (**b**) verbal-learning, and (**c**) levels-of-processing group.

An outline of the experimental setup for the three groups is presented in Fig. 4.

*Social-feedback group.* Upon arrival, participants were told that another, unknown person, would evaluate them based on their self-presentation. In a second condition, a randomly operating computer algorithm was supposedly going to give them feedback. All subjects underwent both "sender" conditions. Condition sequence and word lists were counterbalanced. Importantly, random feedback was presented in both conditions. Participants were instructed to briefly describe themselves in a structured interview in front of a camera. They were informed that the video of their self-description would be presented to another person. During EEG preparation, participants completed a demographic questionnaire and the BDI^50^ and STAI^51^ to characterize the sample. A research assistant left the testing room 15 min ahead of the fictitious feedback to ensure face validity, guiding an "unknown person" to a laboratory room next to the testing room.

Stimuli were presented by software described as "Interactional Behavioral Systems", supposedly allowing instant online communication. Participants were told that the unknown other person would select adjectives describing the participant, which would be subsequently presented. In the other condition, feedback was denoted as random computer feedback. Feedback-adjectives were presented for 1500 ms, after which the participants had to indicate via button-press whether or not they accept this evaluation. After the response, a fixation cross was presented for 2000 to 3500 ms. For each sender, 30 negative, 30 neutral, and 30 positive adjectives (one of the three lists, see Table 4) were presented twice. The desktop environment and stimulus presentation were created using Presentation (www.neurobehavioralsystems.com). Afterwards, participants were debriefed that no social evaluation had taken place and presented a passive viewing run of all 60 negative, 60 neutral, and 60 positive adjectives again to test for any immediate post-processing effects of the manipulation.

Participants were informed that an unrelated test would be conducted in a second session one week later, where an unexpected recognition test took place. All adjectives from the first session and a set of new items were shown for 1500 ms each, followed by an old/new decision and a fixation cross for 1500 to 2000 ms.

*Verbal-learning group.* The very same stimuli and presentation parameters were used. In order to keep the visual input constant, the same background presentation setup (Interactive Behavioral Systems) was used but never referred to. The self-introduction phase was omitted, and no social significance was assigned to the stimulation. Instead, participants were instructed to memorize all adjectives for recognition testing in the next session one week later. Adjectives were presented for 1500 ms, followed by a variable fixation cross presented for 2000 to 3500 ms. To mimic the "social-feedback" experiment, two counterbalanced conditions were presented (block "A" and block "B"), using the same material and number of repetitions (see Fig. 4b, d, Table 4). Condition order was counterbalanced. A passive viewing run finished the experimental session.

*Levels-of-processing group.* The same materials and presentation parameters as in the previous two experiments were used. As in experiment 1, active word evaluation was instructed, but no social context was created. Participants were told to either decide via button press on the self-descriptiveness of the presented words or, in condition two, perform a binary concreteness judgment on the words, classifying them as either abstract or concrete. This closely reflected the encoding trial structure for the social-feedback group. Adjectives were presented for 1500 ms, after which the binary decision was requested. Material and experimental parameters were the same as in the social feedback group (see Fig. 4c, d, Table 4). Again, the session was finished with a passive viewing run.

### EEG recording and analyses

EEG was recorded from 128 BioSemi active electrodes (www.biosemi.com) at 1024 Hz. Two separate electrodes were used as ground electrodes, a Common Mode Sense active electrode (CMS) and a Driven Right Leg passive electrode (DLR), which form a feedback loop to measure the average potential close to the reference in the A/D-box (www.biosemi.com/faq/cms&drl.htm). Four additional electrodes (EOG) measured horizontal and vertical eye movement.

Pre-processing and statistical analyses were performed using BESA (www.besa.de) and EMEGS^53^. Offline, data was re-referenced to an average reference and filtered with a high-pass forward filter of 0.16 (6 db/oct) and a 30 Hz low-pass zero-phase filter (24 db/oct). Filtered data were segmented from 200 ms before word onset until 1500 ms after stimulus presentation. The 200 ms before stimulus onset were used for baseline correction. Eye-movements were corrected using the automatic correction method implemented in BESA^54^. Remaining artifacts were rejected based on an absolute threshold (< 120 µV), signal gradient (< 75 µV/∂T), and low signal (i.e., the *SD* of the gradient, > 0.01 µV/∂T). Noisy EEG sensors were interpolated using a spline interpolation procedure.

On average, 9.5 percent (12.2 in total) of electrodes were interpolated in the social feedback group, and 12 percent of trials were rejected, leading to 53 trials on average. There was no difference in rejected trials concerning encoding or emotion conditions (*Fs* < 1.12, *ps* > 0.302). In the verbal learning group, 3.5 percent (4.5 in total) of the electrodes were interpolated, and 12 percent of trials were rejected, leading to 53 trials on average. There was no difference in rejected trials concerning encoding or emotion conditions (*Fs* < 0.15, *ps* > 0.861). In the LoP group, 7.4 percent (9.4 in total) of electrodes were interpolated, and 24 percent of trials were rejected, resulting in 46 trials on average. There was no difference in rejected trials concerning emotion conditions (*F*_(2,46)_ = 1.54, *p* = 0.225), but a difference concerning encoding conditions (*F*_(1,23)_ = 10.01, *p* = 0.004) with 48 trials on average in the self-reference condition compared to 44 trials in the concreteness condition.

### Statistical analyses

For all data, we calculated three (group: social-feedback, verbal-learning, levels-of-processing) by two (condition: human sender/block A/self-reference, computer sender/block B /concreteness) by three (emotion: positive, negative, neutral) mixed ANOVAs. For memory data, the discrimination index (*P*_*r*_ = hits-false alarms), and the response bias (*B*_*r*_ = false alarms/(1 − *P*_*r*_)) were calculated according to Snodgrass and Corwin's two-high-threshold model^55^. Br values of 0.5 indicate no response bias, while higher values indicate a liberal and lower values a conservative response strategy. Raw data are presented in Supplementary Materials Section A. Furthermore, for the social-feedback and levels-of-processing groups, decision rates (agreement, self-descriptiveness, concreteness) were assessed.

ERP data were analyzed and visualized with EMEGS^53^ and in-house Matlab scripts. Effect sizes were estimated as partial eta-squared^56^. When Mauchly's test indicated a violation of sphericity, degrees of freedom were corrected according to Greenhouse–Geisser. We report uncorrected degrees of freedom but corrected *p*-values and effect sizes. Time windows of interest were segmented from 200 to 400 ms for the EPN. The LPP was scored from 400 to 800 ms and the slow-wave from 800 to 1200 ms. For the EPN, a parieto-occipital sensor region of interest (ROI) of eighteen electrodes was examined (P9, P9h, P7, PO9, PO9h, PO7, I1, OI1, O1, P10, P10h, P8, PO10, PO10h, PO8, I2, OI2, O2). The LPP and the slow-wave were measured at a frontal and a posterior sensor ROI of seventeen electrodes each (frontal: F1, Fz, F2, FFC1, FFC1h, FFCz, FFC2h, FFC2, FC3h, FC1, FC1h, FCz, FC2h, FC2, FC4h, FCC1h, FCC2h; parietal: CCPz, CP3h, CP1, CP1h, CPz, CP2h, CP2, CP4h, CPP1, CPPz, CPP2, P1, Pz, P2, PPO1, PPOz, PPO2).

Finally, exploratory correlations between ERP amplitudes during encoding and memory performance were performed. To this end, mean ERP amplitudes were correlated with different memory indices (Hits and False Alarms (FA)) using JASP (www.jasp.org). We calculated both Bonferroni-corrected inferential and Bayesian Pearson correlation coefficients. For Bayesian analyses, the null hypothesis was specified as a point-null prior (i.e., standardized effect size *δ* = 0). It defined the alternative hypothesis as a Jeffreys-Zellner-Siow (*JZS*) prior, i.e., a folded Cauchy distribution centered around *δ* = 0 with the scaling factor *r* = 0.707. This scaling factor assumes a roughly normal distribution. To assign verbal labels to the strength of evidence, we followed the taxonomy suggested by Jeffreys^57^, labeling Bayes Factors with a BF_10_ of 1 as no evidence, BF_10_ between 1—3 as anecdotal evidence, 3—10 as moderate evidence, 10—30 as strong evidence, 30—100 as very strong evidence, and larger BFs as extreme evidence in favor of the alternative hypothesis.

## Supplementary Information

Supplementary Information.

## References

[1] Ebbinghaus H (1885). Memory: a contribution to experimental psychology. Ann. Neurosci..

[2] Yonelinas AP, Ranganath C, Ekstrom AD, Wiltgen BJ (2019). A contextual binding theory of episodic memory: systems consolidation reconsidered. Nat. Rev. Neurosci..

[3] Edelson M, Sharot T, Dolan RJ, Dudai Y (2011). Following the crowd: brain substrates of long-term memory conformity. Science.

[4] Tavares RM, Mendelsohn A, Grossman Y, Williams CH, Shapiro M, Trope Y, Schiller D (2015). A map for social navigation in the human brain. Neuron.

[5] Dolcos F, Cabeza R (2002). Event-related potentials of emotional memory: encoding pleasant, unpleasant, and neutral pictures. Cogn. Affect. Behav. Neurosci..

[6] Dolcos F, Katsumi Y, Weymar M, Moore M, Tsukiura T, Dolcos S (2017). Emerging directions in emotional episodic memory. Front. Psychol..

[7] Ochsner KN (2000). Are affective events richly recollected or simply familiar? The experience and process of recognizing feelings past. J. Exp. Psychol. Gen..

[8] Kensinger EA, Corkin S (2003). Memory enhancement for emotional words: are emotional words more vividly remembered than neutral words?. Mem. Cognit..

[9] Kensinger EA, Corkin S (2004). Two routes to emotional memory: distinct neural processes for valence and arousal. Proc. Natl. Acad. Sci..

[10] Rogers TB (1977). Self-reference in memory: recognition of personality items. J. Res. Personal..

[11] Sui J, Humphreys GW (2015). The integrative self: how self-reference integrates perception and memory. Trends Cogn. Sci..

[12] Symons CS, Johnson BT (1997). The self-reference effect in memory: a meta-analysis. Psychol. Bull..

[13] Craik FIM, Lockhart RS (1972). Levels of processing: a framework for memory research. J. Verbal Learn. Verbal Behav..

[14] Shrauger JS, Schoeneman TJ (1979). Symbolic interactionist view of self-concept: through the looking glass darkly. Psychol. Bull..

[15] Sharot T, Garrett N (2016). Forming beliefs: why valence matters. Trends Cogn. Sci..

[16] Gutchess A, Kensinger EA (2018). Shared mechanisms may support mnemonic benefits from self-referencing and emotion. Trends Cogn. Sci..

[17] Kissler J, Herbert C, Peyk P, Junghofer M (2007). Buzzwords: early cortical responses to emotional words during reading. Psychol. Sci..

[18] Rugg MD, Curran T (2007). Event-related potentials and recognition memory. Trends Cogn. Sci..

[19] Herbert C, Pauli P, Herbert BM (2011). Self-reference modulates the processing of emotional stimuli in the absence of explicit self-referential appraisal instructions. Soc. Cogn. Affect. Neurosci..

[20] Schindler S, Wegrzyn M, Steppacher I, Kissler J (2015). Perceived communicative context and emotional content amplify visual word processing in the fusiform gyrus. J. Neurosci..

[21] Schupp HT, Flaisch T, Stockburger J, Junghofer M (2006). Emotion and attention: event-related brain potential studies. Prog. Brain Res..

[22] Schupp HT, Stockburger J, Codispoti M, Junghöfer M, Weike AI, Hamm AO (2007). Selective visual attention to emotion. J. Neurosci..

[23] Junghöfer M, Bradley MM, Elbert TR, Lang PJ (2001). Fleeting images: a new look at early emotion discrimination. Psychophysiology.

[24] Friedman D, Johnson R (2000). Event-related potential (ERP) studies of memory encoding and retrieval: a selective review. Microsc. Res. Tech..

[25] Polich J (2007). Updating P300: an integrative theory of P3a and P3b. Clin. Neurophysiol. Off. J. Int. Fed. Clin. Neurophysiol..

[26] Verleger R (2020). Effects of relevance and response frequency on P3b amplitudes: review of findings and comparison of hypotheses about the process reflected by P3b. Psychophysiology.

[27] Bosch V, Mecklinger A, Friederici AD (2001). Slow cortical potentials during retention of object, spatial, and verbal information. Cogn. Brain Res..

[28] Schindler S, Miller GA, Kissler J (2019). Attending to Eliza: rapid brain responses reflect competence attribution in virtual social feedback processing. Soc. Cogn. Affect. Neurosci..

[29] Schindler S, Kruse O, Stark R, Kissler J (2019). Attributed social context and emotional content recruit frontal and limbic brain regions during virtual feedback processing. Cogn. Affect. Behav. Neurosci..

[30] Schindler S, Kissler J (2016). People matter: perceived sender identity modulates cerebral processing of socio-emotional language feedback. Neuroimage.

[31] Fisher RP, Craik FIM (1977). Interaction between encoding and retrieval operations in cued recall. J. Exp. Psychol. [Hum. Learn.].

[32] Rugg MD, Mark RE, Walla P, Schloerscheidt AM, Birch CS, Allan K (1998). Dissociation of the neural correlates of implicit and explicit memory. Nature.

[33] Wang FH (2020). Explicit and implicit memory representations in cross-situational word learning. Cognition.

[34] Hepper EG, Hart CM, Gregg AP, Sedikides C (2011). Motivated expectations of positive feedback in social interactions. J. Soc. Psychol..

[35] Story AL (1998). Self-esteem and memory for favorable and unfavorable personality feedback. Pers. Soc. Psychol. Bull..

[36] Korn CW, Prehn K, Park SQ, Walter H, Heekeren HR (2012). Positively biased processing of self-relevant social feedback. J. Neurosci..

[37] Schindler S, Höhner A, Moeck R, Bruchmann M, Straube T (2021). Let’s talk about each other: neural responses to dissenting personality evaluations based on real dyadic interactions. Psychol. Sci..

[38] Fields EC, Kuperberg GR (2012). It’s all about you: an ERP study of emotion and self-relevance in discourse. Neuroimage.

[39] Bayer M, Ruthmann K, Schacht A (2017). The impact of personal relevance on emotion processing: evidence from event-related potentials and pupillary responses. Soc. Cogn. Affect. Neurosci..

[40] Schacht A, Sommer W (2009). Time course and task dependence of emotion effects in word processing. Cogn. Affect. Behav. Neurosci..

[41] Hinojosa JA, Méndez-Bértolo C, Pozo MA (2010). Looking at emotional words is not the same as reading emotional words: behavioral and neural correlates. Psychophysiology.

[42] L. Rohr, R. Abdel Rahman, Loser! On the combined impact of emotional and person-descriptive word meanings in communicative situations. *Psychophysiology* (2018). doi:10.1111/psyp.13067. 10.1111/psyp.13067 29450885

[43] Herbert C, Junghöfer M, Kissler J (2008). Event related potentials to emotional adjectives during reading. Psychophysiology.

[44] Schwabe L, Bohringer A, Chatterjee M, Schachinger H (2008). Effects of pre-learning stress on memory for neutral, positive and negative words: different roles of cortisol and autonomic arousal. Neurobiol. Learn. Mem..

[45] Zoladz PR, Clark B, Warnecke A, Smith L, Tabar J, Talbot JN (2011). Pre-learning stress differentially affects long-term memory for emotional words, depending on temporal proximity to the learning experience. Physiol. Behav..

[46] Espin L, Almela M, Hidalgo V, Villada C, Salvador A, Gomez-Amor J (2013). Acute pre-learning stress and declarative memory: impact of sex, cortisol response and menstrual cycle phase. Horm. Behav..

[47] Antov MI, Stockhorst U (2018). Women with high estradiol status are protected against declarative memory impairment by pre-learning stress. Neurobiol. Learn. Mem..

[48] P. Light, A.-N. Perret-Clermont, Social context effects in learning and testing. Learn. Think. 136–149 (1991)

[49] Moor BG, van Leijenhorst L, Rombouts SARB, Crone EA, Van der Molen MW (2010). Do you like me? Neural correlates of social evaluation and developmental trajectories. Soc. Neurosci..

[50] M. Hautzinger, F. Keller, C. Kühner, Beck depressions-inventar (BDI-II), Harcourt Test Services, Frankfurt/Main (2006)

[51] C.D. Spielberger, S.J. Sydeman, A.E. Owen, B.J. Marsh, Measuring anxiety and anger with the State-Trait Anxiety Inventory (STAI) and the State-Trait Anger Expression Inventory (STAXI). In: M. E. Maruish (Ed.), Use Psychol. Test. Treat. Plan. Outcomes Assess. 2nd Ed, Lawrence Erlbaum Associates, Mahwah, pp. 993–1021 (1999)

[52] Bradley MM, Lang PJ (1994). Measuring emotion: the self-assessment Manikin and the semantic differential. J. Behav. Ther. Exp. Psych..

[53] Peyk P, De Cesarei A, Junghöfer M (2011). Electro magneto encephalograhy software: overview and integration with other EEG/MEG toolboxes. Comput. Intell. Neurosci..

[54] Ille N, Berg P, Scherg M (2002). Artifact correction of the ongoing EEG using spatial filters based on artifact and brain signal topographies. J. Clin. Neurophysiol..

[55] Snodgrass JG, Corwin J (1988). Pragmatics of measuring recognition memory: applications to dementia and amnesia. J. Exp. Psychol. Gen..

[56] Cohen J (1988). Statistical power analysis for the behavioral sciences.

[57] Jeffreys H (1998). The Theory of Probability.

