# Supplementary material for: Encoding in a social feedback context enhances and biases behavioral and electrophysiological correlates of long-term recognition memory
Source: Sci Rep. 2022 Feb 28;12:3312. doi: 10.1038/s41598-022-07270-9 (PMC8885702; doi:10.1038/s41598-022-07270-9)
Supplement: Supplementary file 1 — Supplementary Information. [file 41598_2022_7270_MOESM1_ESM.docx]

**Encoding in a social feedback context enhances and biases behavioral and electrophysiological correlates of long-term recognition memory**

**SUPPLEMENTARY MATERIALS**

Sebastian Schindler^1,2,3^, Ria Vormbrock^2^ and Johanna Kissler^1,4*^

^1^ Department of Psychology, Bielefeld University

^2^ Institute of Medical Psychology and Systems Neuroscience, University of Muenster

^3^ Otto Creutzfeldt Center for Cognitive and Behavioral Neuroscience, University of Muenster

^4^ Center for Cognitive Interaction Technology (CITEC), Bielefeld University

* Corresponding author

**Correspondence address**

Johanna Kissler

Department of Psychology

Bielefeld University

33501 Bielefeld

Germany

e-mail: [johanna.kissler@uni-bielefeld.de](mailto:johanna.kissler@uni-bielefeld.de)

**Section A: Memory performance one week later**

**Hits and false alarms**

Analyses across the three groups tested for main and interaction effects, including the within factors condition (two levels) and emotion (three levels). Hit rate differed between groups (*F*_(2,65)_ = 32.99, *p* < .001, partial η² = .504; see Supplementary Table S1, Supplementary Figure S1) and was significantly higher in the social-feedback group than both in the verbal-learning (*p* = .002) and the levels-of-processing group (*p* < .001). The verbal-learning group showed significantly more hits than the levels-of-processing group (*p* < .001). There was also an interaction between group and emotion (*F*_(4,130)_ = 4.87, *p* = .001, partial η² = .130) and a three-way interaction between group, condition, and emotion (*F*_(3.61,117.26)_ = 20.53, *p* < .001, partial η² = .387). This three-way interaction was due to condition differences in the levels-of-processing group: The impact of emotional content on the hit rate differed between the levels-of-processing and the concreteness task (see Supplementary Table S1). Experimental groups hardly differed in false alarm rates (*F*_(2,65)_ = 2.51, *p* = .089, partial η² = .072), and there was no substantial interaction of emotion and group (*F*_(3.53,114.64)_ = 2.56, *p* = .050, partial η² = .073) regarding false alarms.

**
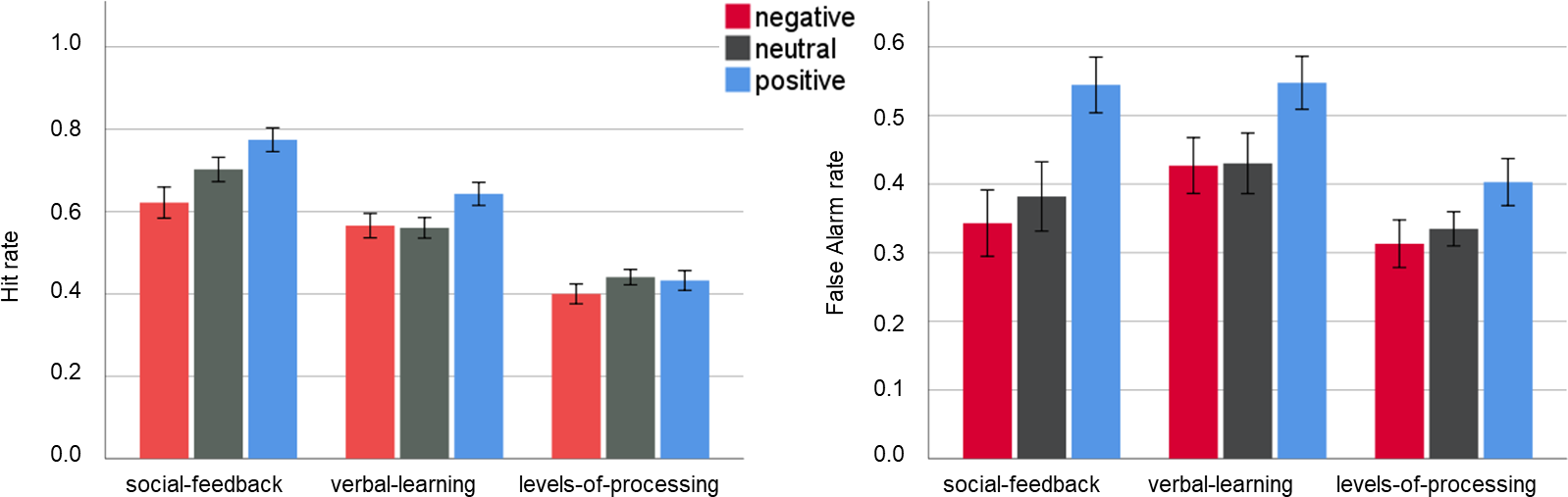
Supplementary Figure S1:** **Behavioral data for the social-feedback, verbal-learning, and levels-of-processing group.** The upper row depicts mean hits and false alarms in per cent per emotional category and group. Error bars depict ±1 Standard Error of the Mean.

**Supplementary Table S1: Hit and false alarm rates for the three groups**

| social-feedback group (*N* = 24) | | | | | | | | | |
| --- | --- | --- | --- | --- | --- | --- | --- | --- | --- |
|  |  |  |  | human feedback | | | computer feedback | | |
|  | condition  *F*_(1,23)_ | emotion  *F*_(2,46)_ | interaction  *F*_(2,46)_ | negative | neutral | positive | negative | neutral | positive |
| Hit rate | 1.12 | **19.93***** | 2.17 | .66  (0.21) | .73  (0.17) | .78  (0.13) | .58  (0.22) | .68  (0.22) | .77  (0.21) |
| FAR |  | **25.41***** |  | .34  (0.24) | .38  (0.25) | .54  (0.20) | .34  (0.24) | .38  (0.25) | .54  (0.20) |
| verbal-learning group (*N* = 21) | | | | | | | | | |
|  |  |  |  | block A | | | block B | | |
|  | condition  *F*_(1,20)_ | emotion  *F*_(2,40)_ | interaction  *F*_(2,40)_ | negative | neutral | positive | negative | neutral | positive |
| Hit rate | 0.07 | **7.27**** | 0.57 | .55  (0.15) | .57  (0.11) | .64  (0.14) | .58  (0.15) | .56  (0.16) | .65  (0.15) |
| FAR |  | **14.11***** |  | .43  (0.19) | .43  (0.20) | .55  (0.18) | .43  (0.19) | .43  (0.20) | .55  (0.18) |
| levels-of-processing group (*N* = 23) | | | | | | | | | |
|  |  |  |  | self-reference task | | | semantic task | | |
|  | condition  *F*_(1,22)_ | emotion  *F*_(2,44)_ | interaction  *F*_(2,44)_ | negative | neutral | positive | negative | neutral | positive |
| Hit rate | 0.04 | 1.93 | **30.06***** | .44  (0.19) | .33  (0.17) | .52  (0.17) | .36  (0.12) | .55  (0.15) | .35  (0.16) |
| FAR |  | **5.72**** |  | .31  (0.17) | .33  (0.12) | .40  (0.17) | .31  (0.17) | .33  (0.12) | .40  (0.17) |

Note: * = *p* < 0.05, ** = *p* < 0.01, *** = *p* < 0.001. Standard deviations appear in parentheses. FAR = False alarm rate. P_r_ = discrimination accuracy, B_r_ = recognition bias. Hits in percent. Significant effects are in bold.

**Section B: ERP differences in the social feedback group during encoding**

Following up on the encoding condiditon x emotion x channel group interaction (*F*_(4,138)_ = 3.48, *p =* .010, η_p_^2^ = .092) within the social feedback group, we explored emotion differences between the two encoding conditions (“human” vs “computer” feedback) for the frontal and parietal ROI. Frontal emotion differences concerned “human feedback” (*F*_(2,46)_ = 4.78, *p =* .013, η_p_^2^ = .172), with a relatively larger positivity for negative and positive compared to neutral feedback (*ps* < .05), positive and negative not differing (*p* = .923) and no effect for the “computer” encoding condition (*F*_(2,46)_ = 0.32, *p =* .731, η_p_^2^ = .014). Parietally, no emotion difference was found in the “human” condition (*F*_(2,46)_ = 1.09, *p =* .344, η_p_^2^ = .045), but there were differences within the computer condition (*F*_(2,46)_ = 13.13, *p <* .001, η_p_^2^ = .363), where an increased positivity for positive feedback compared to both negative and neutral feedback (*ps* < .001) was observed, while negative and neutral did not differ (*p* = .705). No further significant interactions occurred (*Fs* < 2.10*, ps* > .084)*.*


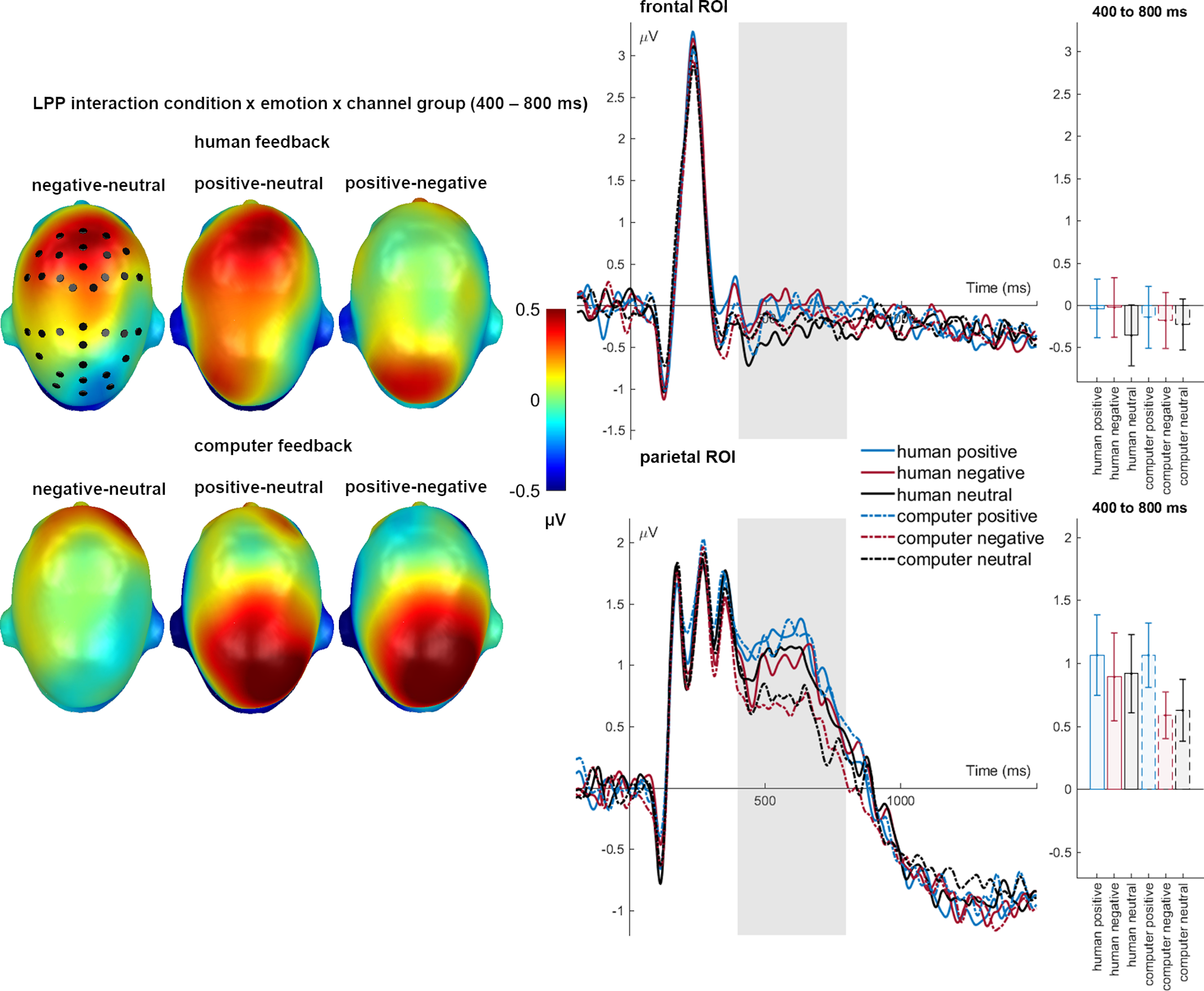
 **Supplementary Figure S2. LPP interaction between encoding condition, emotion, and channel group for the social-feedback group. A)** Scalp topographies depict the mean emotion amplitude differences separately for the encoding conditions (human vs. computer feedback). **B)** Scalp topographies per emotion for differences between the feedback conditions. ERP waveforms show the time course from averaged highlighted sensors. Difference potentials contain 95% bootstrap confidence intervals. For bar charts, error bars show 95% confidence intervals.
